# Metabolic Syndrome Is Associated with Increased Plasma Fibroblast Growth Factor 21 (FGF21) in Postmenopausal Breast Cancer Survivors: Implications for Therapy and Diagnosis

**DOI:** 10.3390/pathophysiology33020031

**Published:** 2026-05-05

**Authors:** Pedro Céspedes, Cristina Buigues, María Dolores Torregrosa, Francisco M. Martínez-Arnau, Omar Cauli, Isabel Trapero

**Affiliations:** 1 Department of Nursing, University of Valencia, 46010 Valencia, Spain; 2 Frailty and Cognitive Impairment Research Group (FROG), University of Valencia, 46010 Valencia, Spain; 3 Chair of Healthy, Active and Participative Aging, University of Valencia, 46010 Valencia, Spain; 4 Medical Oncology Department, Doctor Peset University Hospital, 46017 Valencia, Spain; 5 Department of Physiotherapy, University of Valencia, 46010 Valencia, Spain

**Keywords:** fibroblast growth factor 21, metabolic syndrome, multimodal program, breast neoplasms, postmenopausal women, exercise intervention, aromatase inhibitors, hypertension, hyperglycemia, biomarkers

## Abstract

Background: Fibroblast growth factor 21 (FGF21) is a peptide hormone that is synthesized by several organs and regulates energy homeostasis, including reducing fat mass and lowering hyperglycemia, insulin resistance and dyslipidemia. It also increased metabolic syndrome (MS) and cardiovascular risk in breast cancer (BC) survivors treated with aromatase inhibitors (AIs) aimed at reducing cancer recurrence. We evaluated whether blood FGF21 concentration is associated with MS and its five criteria in postmenopausal women treated with AIs, and whether this persists after multimodal interventions that reduce MS. Methods: A quasi-experimental longitudinal study in 31 postmenopausal women with localized BC on Ais, assessed via a 12-week multimodal program. Their MS was evaluated per the NCEP-ATP III guidelines (waist circumference, blood pressure, fasting glucose, triglycerides, HDL-cholesterol). Plasma FGF21 was measured pre/post-intervention via fasting blood samples, centrifugation, and ELISA assay. Results: Pre-intervention FGF21 median: 377.62 pg/mL (38.40–1616.42). Plasma FGF21 concentrations positively correlated with MS criteria number pre- and post-intervention (all *p* < 0.05). Linear regression confirmed pre-intervention MS criteria (β = 127.262, *p* = 0.006) and antihypertensive drugs as predictors of post-FGF21 levels. Analysis of individual MS criteria revealed significant associations with blood pressure (*p* = 0.028 and *p* = 0.022 for systolic and diastolic pressure, respectively) and fasting glucose changes (*p* = 0.008). Conclusions: Plasma FGF21 may act as a biomarker for monitoring exercise-based interventions which reduce MSs, particularly hypertension and hyperglycemia, in AI-treated BC survivors.

## 1. Introduction

The absolute number of breast cancers diagnosed has increased in recent decades due to population growth, an aging population, exposure to risk factors and increased early detection [1,2,3]. The most common type of breast cancer is characterized by an increased expression of estrogen and/or progestogen receptors. Adjuvant drug therapy with anti-estrogens is recommended after surgical treatment for many women with localized, hormone receptor-positive breast cancer. In postmenopausal women, these are aromatase inhibitors, an enzyme that converts androgens into estrogens, and are therefore anti-estrogenic therapies. This adjuvant therapy is used for 5–10 years, with the aim of preventing recurrence and increasing cancer-free survival. As a chronic drug treatment, it has various adverse effects, including metabolic syndrome (MS). MS is a common syndrome in the general population, characterized by a range of metabolic factors that increase the risk of cardiovascular disease, type 2 diabetes mellitus (DM2), and other diseases, including various types of cancer [4,5,6]. The pathophysiological factors involved in metabolic syndrome are insulin resistance, excess abdominal fat, atherogenic dyslipidemia, endothelial dysfunction, genetic susceptibility, high blood pressure, hypercoagulability, and chronic stress. The parameters used to define this syndrome are hypertriglyceridemia, low high-density lipoprotein (HDL) cholesterol, insulin resistance, high blood pressure, and central obesity [4,5]. The risk of cardiovascular disease associated with MS is a particularly important factor in women with breast cancer, as they have a significantly higher risk of cerebrovascular disease than women without cancer [7,8,9]. Various studies [10,11,12] have specifically demonstrated how the impact of aromatase inhibitors on metabolic and cardiovascular health in patients diagnosed with breast cancer increases the risk of cardiovascular events. Modifications of habits and lifestyles that are considered unhealthy or very unhealthy are therefore a decisive factor in women undergoing aromatase inhibitor treatments in order to prevent metabolic and cardiovascular events and further limit cancer recurrence, since both obesity and hyperinsulinemia associated with insulin resistance are considered risk factors for hormone-dependent breast cancer [13,14]. Fibroblast growth factor 21 (FGF21) is a polypeptide produced mainly in the liver [15] which has been identified as an endocrine and metabolic hormone due to its powerful effect on lipid and glucose metabolism, as well as on insulin sensitivity and energy balance [16]. An animal study showed that FGF21 had favorable effects by reducing serum glucose and triglyceride levels and improving lipoprotein profiles in genetically compromised FGF transgenic mice and primates [17,18]. Administration of FGF21 analogs has demonstrated significant reductions in fasting insulin levels, body weight, and total cholesterol, supporting its translational potential in clinical practice [19,20]. However, there is growing evidence that FGF21 is a potential marker of metabolic diseases in humans. Epidemiological studies have revealed that higher serum FGF21 levels are an independent predictor of MSs in Asian individuals, and that FGF21 levels are significantly higher among prediabetic and diabetic patients and may predict the development of diabetes in a Chinese population [21]. Circulating concentrations of FGF21 are positively associated with parameters in Japanese and Chinese patients with DM2 [22,23] and with the onset of MS and DM2 in patients of European descent [24]. Beyond metabolic disease, FGF21 has recently gained interest in breast cancer studies. At diagnosis, women with breast cancer display significantly elevated serum FGF21 levels compared with age-matched healthy controls [25]. We assessed whether blood FGF21 is associated with metabolic syndrome and its relationship with each of the five criteria of this syndrome in breast cancer under hormonal therapy with AIs. We also analyzed whether this association persists after a multimodal intervention known to decrease MS as previously reported [26], and examined which of the five criteria of MS correlate with the FGF21 changes observed after this intervention. Multivariate analyses were performed to identify the role of independent factors, such as age, previous chemotherapy, time under aromatase inhibitor therapy, body mass index and polypharmacy, to predict changes in plasma FGF21 levels.

## 2. Materials and Methods

### 2.1. Study Design and Sample Size

This is a quasi-experimental longitudinal study based on a 12-week physical exercise and health education program (as described in [26]) aimed at improving MSs in postmenopausal women who had localized breast cancer and were receiving adjuvant hormone therapy to prevent recurrence. A convenience sampling method was used for this study. Based on published data regarding the prevalence of MSs among Spanish older women, we estimated an a priori prevalence of approximately 40% in our study sample. A total of 90 women met the inclusion criteria at the oncology unit. With an alpha risk set at 0.05 and a statistical power of 0.8 using a two-tailed test, a minimum of 46 participants was required to detect a statistically significant difference. This sample size estimation assumed an initial MS prevalence of approximately 40% in the study population and an anticipated reduction to 20% following the intervention. Taking into account calculating the sample size based on a good correlation between FGF21 concentration in plasma and the number of MS criteria, the sample size needed to detect a correlation of r = 0.5 with a two-sided α = 0.05 and 80% power; therefore, the minimum sample size is minimum n ≈ 30 patients.

Ethical approval for this project was granted by the ethics committee of Dr. Peset University Hospital in Valencia, Spain (protocol number 102.22; approval date: 26 October 2022). All physical assessments and blood analyses were conducted at the Medical Oncology Department of the same hospital. The study period extended from September 2023 to April 2024. Participants were excluded if they had cognitive impairment or insufficiently controlled mental illness, given that such conditions could hinder their comprehension of the health education sessions. Likewise, individuals with physical or functional limitations preventing them from carrying out the exercise components of the program were not eligible for inclusion.

### 2.2. Socio-Demographic and Breast Cancer-Related Variables

Data collection included socio-demographic variables, namely age and marital status, along with clinical variables, including the date of breast cancer diagnosis, whether chemotherapy or radiotherapy had previously been administered, polypharmacy, and the date anti-estrogen therapy began.

### 2.3. Measurement of Metabolic Syndrome

Metabolic syndrome was evaluated based on its five constituent criteria—waist circumference, triglycerides, HDL cholesterol, systolic and/or diastolic blood pressure, and fasting glucose—in accordance with the third edition of the NCEP-ATP III guidelines [4]. Abdominal circumference was assessed using a Dioche anatomical tape measure, and blood pressure was recorded with an OMRONX3 digital arm monitor (Omron Corporation, Kyoto, Japan). The remaining parameters (fasting blood glucose, triglycerides, and HDL) were derived from blood analyses that also included a complete blood count and a biochemistry panel. Blood samples were drawn under fasting conditions between 7:30 a.m. and 9 a.m., collected in EDTA-containing vacutainers, and processed at the hospital laboratory. The complete blood count additionally served to confirm the absence of anemia among the participants. Anthropometric measurements were obtained using a Tanita bioelectrical impedance scale (InnerScan BC545N, Tokyo, Japan). Consistent instrumentation was maintained across all participants at both pre- and post-intervention assessments. In line with NCEP-ATP III definitions [4], MS was diagnosed when at least 3 of the 5 criteria were fulfilled. Furthermore, to evaluate the effect of the multimodal program on MS, MS status was dichotomized into two groups: women who showed improvement (i.e., a reduction in the number of MS criteria met after the intervention relative to baseline) versus women whose number of MS criteria remained unchanged or increased following the intervention.

### 2.4. Measurement of Plasma FGF21 Concentration

For FGF21 measurement, an additional venous blood sample (1 mL) was obtained from each participant through venipuncture of an antecubital vein and collected in vacutainer tubes containing ethylenediaminetetraacetic acid (EDTA). They were then centrifuged at 1500× *g* for 10 min at room temperature to extract the plasma, which was stored at −80 °C.

FGF21 plasma concentrations (pg/mL) were determined using a dedicated ELISA kit (Abcam^®^, catalog number ab222506, Cambridge, UK), which offered a sensitivity of 3.3 pg/mL and a detection range of 31.25–2000 pg/mL. The assay was performed in accordance with the manufacturer’s protocol.

### 2.5. Statistical Analysis

First of all, descriptive statistical tests were conducted. Continuous variables were expressed as means and standard deviations, whereas categorical variables were reported as frequencies and percentages. Normality was assessed through the Shapiro–Wilk test, complemented by visual inspection of histograms and Q–Q plots; homogeneity of variances was tested using Levene’s test. Correlations between quantitative variables were examined by means of Spearman’s rank correlation coefficient for non-normally distributed data and Pearson’s correlation coefficient for normally distributed data. Pre- to post-intervention changes were analyzed with the paired Student’s *t*-test when data met parametric assumptions, and the Wilcoxon signed-rank test otherwise. Differences in dichotomous categorical variables were assessed using McNemar’s test, while the marginal homogeneity test was used for categorical variables comprising more than two categories.

Two-group comparisons were performed using the Mann–Whitney U test for non-parametric data or Student’s *t*-test for parametric data. When three groups were compared, the Kruskal–Wallis test was applied for non-parametric data and one-way ANOVA for parametric data.

Linear regression analysis was performed to explore the associations with post-intervention FGF21 blood concentrations and various predictor variables that demonstrated statistical significance in bivariate analyses and/or possessed robust clinical evidence: age, number of MS criteria pre-intervention, receipt of chemotherapy, duration of hormone therapy, time since breast cancer diagnosis, number of daily medications, use of antihypertensive, antihyperlipidemic, and/or anti-diabetic medications, and post-intervention body mass index (BMI).

Unstandardized and standardized beta coefficients, 95% confidence intervals (95% CIs), and *p*-values were calculated for each predictor. Statistical significance was set at *p* < 0.05 for all tests.

The data analyses were performed using SPSS, version 29.0 (SPSS Inc., Chicago, IL, USA), licensed to the University of Valencia.

## 3. Results

### 3.1. Sociodemographic and Clinical Data

Thirty-one patients with breast cancer were evaluated at baseline and after 3 months of intervention. The mean age was 66.10 ± 0.87 years. All patients were undergoing AI treatments. None of the patients presented with distant metastases, as confirmed by review of the staging imaging reports available for each participant together with the corresponding clinical reports from the treating oncologists. At the time of inclusion in the study, TNM stage was local stage I in (48.4%) of patients and early regional stage II in (51.6%). All the patients had already undergone surgery before participating in the program (87.1% had breast-conserving surgery) and had completed adjuvant chemotherapy and/or radiotherapy treatments, as necessary (41.9% received chemotherapy and all but one received radiotherapy). Nineteen (61.3%) of the women were married, while 48.4% of the women met the criteria for polypharmacy (five or more medications daily), and the participants’ mean BMI was 29.51 ± 0.87 (SEM) (range 21.7–39), with seven of the women (22.6%) meeting the normal weight criteria (18.5–24.9). All the sociodemographic and clinical data are shown in Table 1. Regarding specific drugs prescribed to treat MS criteria, 16 women (51.6%) were taking anti-hypertensive drugs, 19 women (61.3%) drugs for dyslipidemia, and 6 (19.4%) anti-diabetic drugs.

### 3.2. Metabolic Syndrome and Its Relationship with Socio-Demographic and Clinical Characteristics

There were no statistically significant differences in women’s age (*p* = 0.699, Mann–Whitney test), time on hormone therapy (*p* = 0.611, Mann–Whitney test), or BMI (*p* = 0.670, Student’s *t*-test) between the groups with and without pre-intervention criteria for sudden death. Similarly, these variables were not quantitatively correlated with the number of criteria for sudden death before the intervention, i.e., with neither age (Spearman’s rho = −0.105; *p* = 0.460), time on hormone therapy (Spearman’s rho = −0.115; *p* = 0.410), nor BMI (Spearman’s rho = 0.180; *p* = 0.197). However, there were significant differences in the number of medications taken (*p* = 0.036, Mann–Whitney test). MS was not associated with prior chemotherapy (*p* = 0.056, Mann–Whitney U test), prior radiotherapy (*p* = 0.645, Mann–Whitney U test), or dichotomized surgery (*p* = 0.887, Mann–Whitney U test).

### 3.3. Changes in Metabolic Syndrome After Intervention

Following the intervention, a significant reduction was observed in the mean number of MS criteria met in the sample, which fell from 2.50 ± 1.53 (SEM) (range 0 to 3) to 1.80 ± 1.31 (*p* = 0.002). Similarly, statistically significant reductions in individual MS parameters were obtained in the number of patients exceeding the blood pressure cut-off point (27 (87.1%) before and 15 (48.4%) after the intervention; *p* = 0.001) and abdominal circumference (27 (87.1%) before and 20 (64.5%) afterwards; *p* = 0.001) for MS criteria. Significant differences were also found in the mean values of these two individual parameters as quantitative variables for systolic blood pressure (145.39 ± 4.19 (SEM) before and 132.61 ± 4.01 after the intervention *p* < 0.001), diastolic blood pressure (96.32 ± 2.35 (SEM) before and 85.39 ± 2.11 after the intervention *p* < 0.001), and abdominal circumference (99.71 ± 1.82 (SEM) before and 97.10 ± 1.70 after the intervention *p* < 0.001). Conversely, no significant differences were found after the intervention in the parameters of fasting glucose, triglycerides, and HDL (neither with cut-off points nor as quantitative variables).

### 3.4. Relationship Between Metabolic Syndrome and FGF21 Blood Concentration

The median concentration of FGF21 in blood in all the participants before the intervention was 377.62 pg/mL (range 38.40–1616.42). These values are within the range reported in other published articles [27,28,29,30]. The number of MS criteria met in women before the intervention was significantly and positively correlated with pre-intervention FGF21 blood hormone concentrations (Spearman’s rho = 0.408; *p* = 0.023). Similarly, the number of MS criteria met in patients after the intervention was positively correlated with post-intervention FGF21 blood hormone concentrations (Spearman’s rho = 0.468; *p* = 0.009). A significant direct correlation was also observed between the number of MS criteria met before the intervention and post-intervention FGF21 blood hormone concentrations (Spearman’s rho = 0.633; *p* < 0.001).

**Figure 1:**
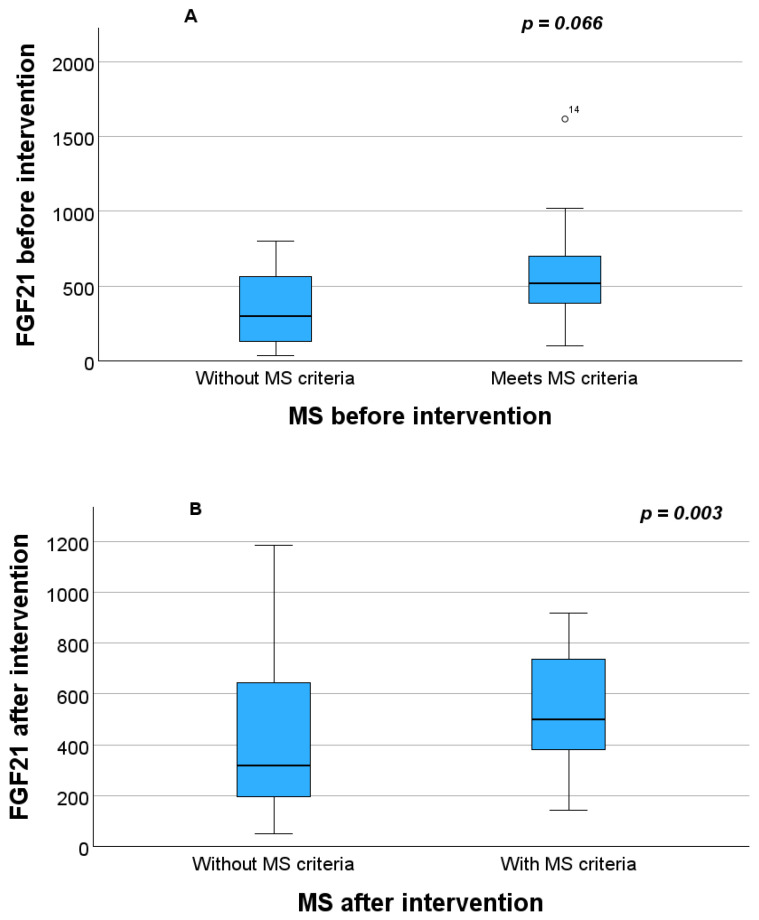
Differences in FGF21 between groups with and without MS criteria before (**A**) and after the intervention (**B**). On the boxplots shown in the Figure the outliers are identified (“out” values (small circle)).

At baseline (before the intervention), the differences in blood FGF21 concentration between the women meeting MS criteria (three or more parameters) (median 520.21 pg/mL, range 98.99–1616.42) and those who did not (median 301.11 pg/mL, range 38.40–80.39) were not statistically significant (*p* = 0.066) (Figure 1A). However, statistically significant differences in FGF21 concentration were found between women with and without MSs after the intervention (median 590.48 pg/mL, range 199.02–1182.99 with MS and median 217.49 pg/mL, range 50.43–811.17 not meeting MS criteria; *p* = 0.003 (Figure 1B)).

Prior to the intervention, abdominal circumference (Spearman’s rho = 0.491; *p* = 0.005), and triglycerides (Spearman’s rho = 0.415; *p* = 0.02) showed significant positive correlations with pre-intervention blood FGF21 levels. Statistically significant positive correlations (Spearman’s rho = 0.486 *p* = 0.006) were also obtained between BMI and FGF21 concentration before the intervention. In contrast, systolic blood pressure (Spearman’s rho = 0.217; *p* = 0.242), diastolic blood pressure (Spearman’s rho = 0.094; *p* = 0.615), fasting glucose (Spearman’s rho = 0.329; *p* = 0.070), and HDL (Spearman’s rho = −0.301; *p* = 0.100) did not exhibit statistically significant associations with pre-intervention blood FGF21 levels. Furthermore, after the intervention, the variables systolic blood pressure (Spearman’s rho = 0.375; *p* = 0.041), fasting glucose (Spearman’s rho = 0.495; *p* = 0.005), abdominal perimeter (Spearman’s rho = 0.401; *p* = 0.028), and triglycerides (Spearman’s rho = 0.440; *p* = 0.015) were significantly and positively correlated with FGF21 levels after the intervention. Diastolic blood pressure (Spearman’s rho = 0.375; *p* = 0.094) and HDL (Spearman’s rho = −0.365; *p* = 0.053) were not.

### 3.5. Multivariate Analyses

A linear regression analysis was performed to explore the associations between FGF21 blood concentrations after the intervention and various predictor variables that showed statistical significance in the bivariate analysis and/or presenting strong clinical evidence: age, the number of MS criteria before the intervention, whether or not the patient had received chemotherapy, the duration of hormone therapy, the time since BC diagnosis, the number of daily medications, whether or not they were taking medication for blood pressure, hyperlipidemia and/or diabetes, and BMI after the intervention (Table 2). This linear regression showed a significant positive association between the number of MS criteria the women met before the intervention and their FGF21 blood concentration after the intervention (*p* = 0.006, unstandardized beta coefficient = 127.262, 95% CI 41.603–212.920). Patients meeting more MS criteria before the intervention were therefore associated with higher FGF21 blood concentration levels after the intervention. In addition, the use of antihypertensive (*p* = 0.018, unstandardized beta coefficient = 297.277, 95% CI 56.426–538.128) and lipid-lowering drugs (*p* = 0.019, unstandardized beta coefficient = −282.826, 95% CI −513.787–−513.787) also showed a relationship with the dependent variable.

### 3.6. Relationship Between the Progression of FGF21 and Metabolic Syndrome After Intervention

**Figure 2:**
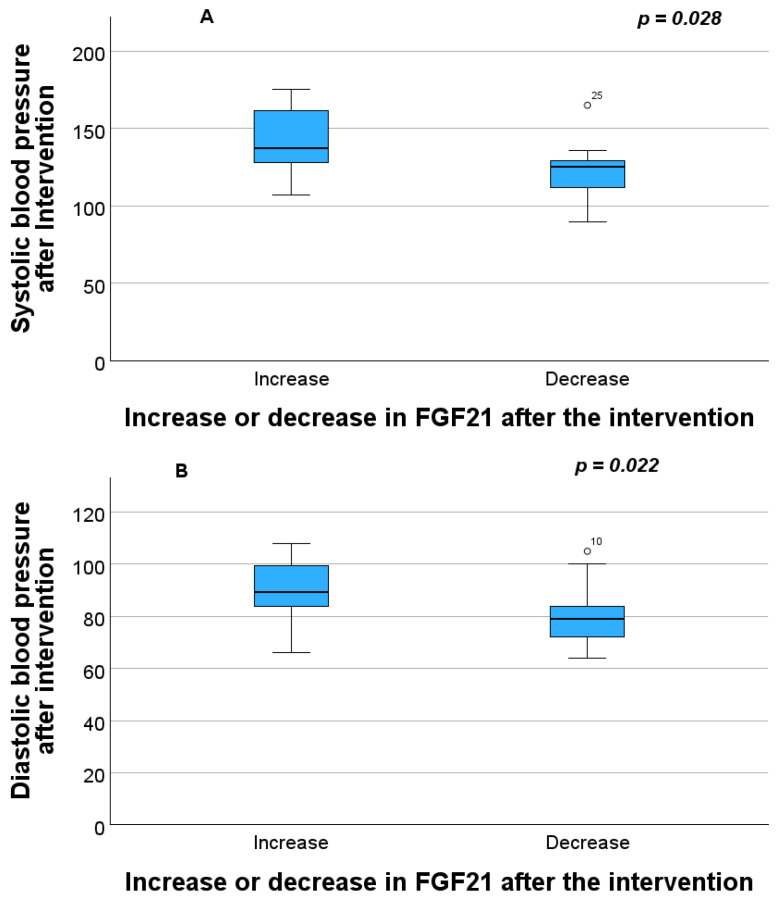

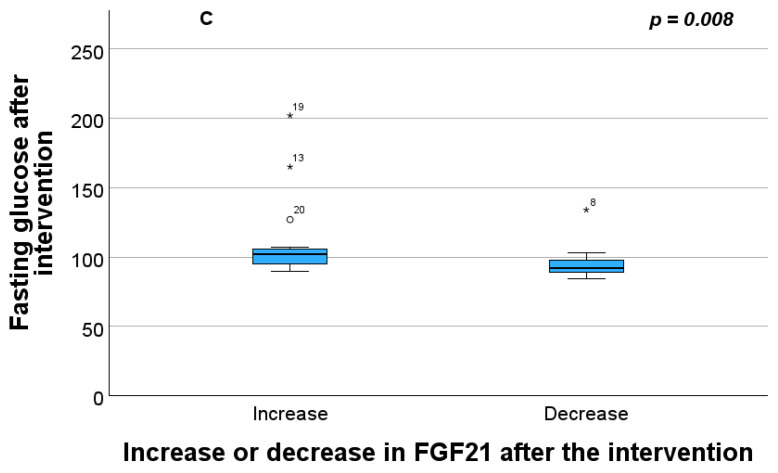
Differences between systolic blood pressure (**A**), diastolic blood pressure (**B**) and fasting glucose (**C**) after the intervention in patients whose FGF21 biomarker increased after the intervention and those group whose FGF21 decreased. On the boxplots shown in the Figure the outliers are identified (“out” values (small circle) and “far out” and “Extreme values” (marked with a star)).

No significant differences were found in the mean FGF21 value between before (median 377.62 pg/mL, range 38.40–1616.42) and after the intervention (median 424.67 pg/mL, range 50.43–1182.99). This was also the case for the number of MS parameters between the group with increased FGF21 concentration (median two parameters, range 0–5 parameters) and those without (median 1 parameter, range 0–4 parameters). In regard to the individual criteria that comprise MS, statistically significant differences were found in systolic blood pressure (*p* = 0.028) (Figure 2A), diastolic blood pressure (*p* = 0.022) (Figure 2B), and glucose (*p* = 0.008) (Figure 2C) values between the patients whose FGF21 concentration levels increased after the intervention compared to baseline and the group in which this concentration decreased after the intervention (Table 3). No differences were found in abdominal perimeter, triglycerides, or HDL (Table 3). In the qualitative fasting glucose variable (applying the cut-off point for MS) statistically significant differences in FGF21 concentration were also observed after the intervention (*p* = 0.018).

The difference in FGF21 concentration between the patients before and after the intervention and the percentage change in FGF21 concentration did not correlate significantly with the number of MS criteria met after the intervention (Spearman’s rho = 0.134; *p* = 0.482 and Spearman’s rho = 0.177; *p* = 0.35, respectively). However, when considering the individual MS criteria, significant direct correlations were found between these variables (the difference in FGF21 concentration levels between patients before and after the intervention, and the percentage change in FGF21 concentration) and the values for systolic blood pressure (SBP), diastolic blood pressure (DBP), and fasting glucose after the intervention. No significant correlations with abdominal circumference, triglycerides or HDL cholesterol were found.

## 4. Discussion

Breast cancer treatment for estrogen receptor-positive tumors in postmenopausal women is based on anti-estrogen therapy with AIs, which increases the risk of developing or worsening MS [31,32,33,34]. In addition, the risk of developing high blood pressure, dyslipidemia, insulin resistance, or diabetes mellitus increases in postmenopausal women, even without the use of AIs [35].

Fibroblast growth factor 21 (FGF21) is a peptide hormone that is synthesized by several organs, predominantly expressed in the liver, pancreas, and white adipose tissue, and regulates energy homeostasis [36,37,38]. It plays a key role in regulating glucose and lipid metabolism, insulin sensitivity, energy balance, and adipose tissue function. Studies have highlighted its potential as an insulin-sensitizing and lipolysis-suppressing molecule that protects against systemic metabolic dysregulation, especially in patients with fatty liver disease. High blood concentrations of FGF21 have been associated with obesity and related disorders such as hepatic steatosis and DM2. FGF21 levels correlate proportionally with body mass index and are closely related to visceral obesity [39]. According to these findings, we cross-sectionally observed the association between FGF21 concentration and lipid/fat content (with triglyceride concentration, BMI, and abdominal circumference) at baseline, and with triglyceride concentration and abdominal circumference after the intervention. In our study, we replicated the association between plasma FGF21 concentration and the number of MS criteria at two time points, at baseline (i.e., before the intervention) and after an intervention based on physical exercise and health education workshops aimed at reducing MS severity in breast cancer survivors undergoing AI treatments [26]. The association between FGF21 in blood and MS has been reported in cross-sectional and longitudinal studies. Baseline FGF21 levels are elevated in participants with prevalent MSs [40]. The correlation between serum FGF21 level and the incidence of MS was also found in a prospective study performed in adults in Korea and China [23,41], in Germany [42], and in mixed ethnic population in the United States [40].

The fact that the association between FGF21 concentration in blood and the number of fulfilled criteria for MS remain constant under AIs, as occurs in the general population, suggests that its measurement could in future studies predict the incidence of coronary artery disease, diabetes mellitus and renal progression in diabetes and all-cause and cardiovascular mortality in the general population [43] as well as among breast cancer survivors: these patients also have an increased risk of those comorbidities under this pharmacological treatment aimed at reducing cancer recurrence [12,44,45,46,47].

Blood concentrations of FGF21 are associated with both systolic and diastolic blood pressure [29,48,49,50]. The association persisted after adjusting for sex, age, and different confounders such as BMI, hypercholesterolemia, obesity, diabetes, alcohol consumption, smoking, and antihypertensive treatment [49,50].

Two criteria of metabolic syndrome have been linked to FGF21, with changes in FGF21 levels following the intervention, according to multivariate analysis of the effects of the intervention that reduces metabolic syndrome in these patients [26].

The association of FGF21 with blood pressure and hypertension in our study supports the previously proposed concept of FGF21 resistance in the regulation of blood pressure in patients with hypertension [50]. FGF21 is involved in the regulation of blood pressure and contributes to its reduction [50,51]. However, circulating FGF21 levels are significantly elevated in people with hypertension. In our study, women whose FGF21 concentration was lower after the intervention had lower systolic and diastolic blood pressure values. Paradoxical changes in FGF21 levels have also been observed in previous studies [50,52]. FGF21 has been observed to stimulate glycogen production in the liver and induce lipolysis in white adipose tissue during fasting and starvation. Surprisingly, instead of having deficient levels, obese and diabetic db/db mice and obese individuals show elevated levels of FGF21 [27]. Resistance to FGF21 is promoted by central resistin via the toll-like receptor 4, through downregulation of hypothalamic FGF21 expression and hypothalamic and peripheral expression of its receptor components [53].

FGF21 is related to glucose homeostasis and various studies have reported an increase in FGF21 in the blood of individuals with diabetes who also have overweight/obesity [19,54,55]; furthermore, plasma FGF21 concentrations are positively associated with the HOMA-IR (Homeostasis Model Assessment—Insulin Resistance) index [56]. In line with these previous findings, patients with decrease in FGF21 concentrations after the intervention have significant lower glycemia compared to those who had an increase in FGF21 after the intervention.

Both eating and fasting signals paradoxically and independently control the expression of the human FGF21 gene. These regulatory systems imply that nutritional crises, such as famine and overfeeding, raise human FGF21 [37,57].

There are several significant limitations to the current investigation. First, for a common malignancy like breast cancer, the sample size was comparatively modest. Additional studies are warranted to confirm the results of this report. However, we believe that the study offers directions for further research in this fascinating area of long-term side effects of anti-estrogenic therapy. The impact of prior chemotherapy regimens did not significantly alter the study’s results; however, we cannot completely rule out the possibility that a greater number of patients with prior chemotherapy might have contributed to certain variations in the study’s results. The study has an important limitation about the reduced sample size. The number of patients should have been roughly 60–65 women when determining the sample size for multiple regression analysis with several predictors, which would have decreased the generalizability of the current findings. Because the analyses in this study were exploratory and based on a small number of pre-specified hypotheses concerning the relationship between plasma hormone concentrations and physical activity measures, multiple testing adjustment was not used. As a result, results are reported unadjusted but should be evaluated cautiously, and independent cohort replication is required. However, the study’s longitudinal methodology and the fact that significant correlations for FGF21 levels in blood and number of MS criteria were found at two time points, with an elapsed time between them of 12 weeks, makes it possible to reinforce the strength of this association despite the exploratory nature of this research study.

Additional studies are warranted to understand the relationship between FGF-21 changes and individual components of metabolic syndrome and their adverse outcomes.

## 5. Conclusions

Our findings suggest that plasma FGF21 concentration could be a biomarker that can be measured to monitor the changes induced by physical exercise-based interventions that reduce MS and two criteria in particular, i.e., arterial hypertension and hyperglycemia, in breast cancer survivors receiving aromatase inhibitors to prevent breast cancer relapse.

## Figures and Tables

**Table 1:** Demographic and clinical characteristics.

Variables	Total Number of Women Starting the Program (n = 31)
Demographic Variables	
Age, years	
Mean (SD)	66.10 ± 0.87
Range	60–78
Level of education	
Primary	9 (29%)
Secondary	17 (54.8%)
University studies	5 (16.2%)
Marital status	
Married	19 (61.3%)
Widowed	3 (9.7%)
Divorced	3 (9.7%)
Single	6 (19.3%)
Clinical variables	
BC Stage at diagnosis	
I	15 (48.4%)
II	16 (51.6%)
Previous chemotherapy	
Yes	13 (41.9%)
No	18 (58.1%)
Previous radiotherapy	
Yes	30 (96.8%)
No	1 (3.2%)
Type of surgery	
Conservative	27 (87.1%)
Mastectomy	4 (12.9%)
Polypharmacy criteria	
Yes	16 (51.6%)
No	15 (48.4%)
BMI classification	
Normal weight	7 (22.6%)
Overweight	8 (25.8%)
Grade I Obesity	12 (38.7%)
Grade II Obesity	4 (12.9%)

BC: Breast Cancer.

**Table 2:** Multivariable linear regression model: association of FGF21 concentration after the intervention with predictive clinical variables.

Variables	Unstandardized Coefficient		Standardized Beta Coefficient	*t*	*p*-Value	95% CI	
	B	Std Error				LL	UL
Age	−16.401	10.885	−0.267	1.507	0.148	−39.182	6.381
Number of MS criteria met before the intervention	127.262	40.926	0.518	3.110	0.006	41.603	212.920
Receiving chemotherapy (ref: No)	−20.221	100.850	−0.034	−0.201	0.843	−231.302	190.861
Time since BC diagnosis	2.702	3.318	0.285	0.814	0.426	−4.243	9.648
Time (months) undergoing HT treatment	−1.569	3.811	−0.140	−0.412	0.685	−9.547	6.408
Number of different medications taken	26.667	17.818	0.306	1.497	0.151	−10.627	63.961
Antihypertensive medication (ref: No)	297.277	115.073	0.501	2.583	0.018	56.426	538.128
Lipid-lowering medication (ref: No)	−282.826	110.348	−0.460	−2.563	0.019	−513.787	−513.787
Medication for diabetes (ref: No)	−151.330	132.962	−0.205	−1.138	0.269	−429.623	126.962
BMI	−13.243	10.814	−0.206	−1.225	0.236	−35.876	9.390

(BMI: Body mass index; BC: Breast Cancer; HT: Hormonotherapy; MS: Metabolic syndrome; B: Unstandardized beta coefficient; Std Error: Standard Error; LL: lower limit; UL: upper limit.) R = 0.810, R squared = 0.657, Adjusted R squared = 0.476. Anova F-Test: 3.633. Anova *p*-value = 0.008.

**Table 3:** Analysis of systolic blood pressure, diastolic blood pressure, abdominal perimeter, fasting glucose, triglycerides and HDL after the intervention in patients whose FGF21 concentration increased after the intervention, and the patients whose levels decreased.

Variables	Patients with Increased FGF21 Concentration After the Intervention Mean (SD)	Patients with Decreased FGF21 Concentration After the Intervention Mean (SD)	*p*-Value *
Systolic blood pressure after the intervention	140.94 (23.18)	122.64 (18.28)	0.028
Diastolic blood pressure after the intervention	89.44 (11.13)	80.57 (11.35)	0.022
Abdominal perimeter after the intervention	96.28 (8.68)	99.143 (9.73)	0.355
Fasting glucose after the intervention	111.13 (30.33)	95.43 (12.17)	0.008
Triglycerides after the intervention	110.69 (50.35)	114.43 (57.48)	0.951
HDL after the intervention	54.25 (10.53)	55.29 (12.21)	0.886

* Obtained with the Mann–Whitney test. HDL (high-density lipoprotein).

## Data Availability

The data presented in this study are available from the corresponding author on request due to ethical restrictions.
